# Progress in Mathematical Modeling of Gastrointestinal Slow Wave Abnormalities

**DOI:** 10.3389/fphys.2017.01136

**Published:** 2018-01-15

**Authors:** Peng Du, Stefan Calder, Timothy R. Angeli, Shameer Sathar, Niranchan Paskaranandavadivel, Gregory O'Grady, Leo K. Cheng

**Affiliations:** ^1^Auckland Bioengineering Institute, University of Auckland, Auckland, New Zealand; ^2^Department of Surgery, University of Auckland, Auckland, New Zealand; ^3^Department of Surgery, Vanderbilt University, Nashville, TN, United States

**Keywords:** slow wave, GI, multi-scale modeling, arrhythmias, Electrophysiology

## Abstract

Gastrointestinal (GI) motility is regulated in part by electrophysiological events called slow waves, which are generated by the interstitial cells of Cajal (ICC). Slow waves propagate by a process of “entrainment,” which occurs over a decreasing gradient of intrinsic frequencies in the antegrade direction across much of the GI tract. Abnormal initiation and conduction of slow waves have been demonstrated in, and linked to, a number of GI motility disorders. A range of mathematical models have been developed to study abnormal slow waves and applied to propose novel methods for non-invasive detection and therapy. This review provides a general outline of GI slow wave abnormalities and their recent classification using multi-electrode (high-resolution) mapping methods, with a particular emphasis on the spatial patterns of these abnormal activities. The recently-developed mathematical models are introduced in order of their biophysical scale from cellular to whole-organ levels. The modeling techniques, main findings from the simulations, and potential future directions arising from notable studies are discussed.

## Gastrointestinal electrophysiological definitions and disorders

Cyclical contractions of the gastrointestinal (GI) tract are initiated and coordinated by rhythmic, propagating bioelectrical events, termed slow waves (Szurszewski, 1998). Abnormal slow wave activity has long been observed to occur in the gut, and their putative causation of gastrointestinal symptoms and disorders remains an area of significant interest (Lammers, 2013; O'Grady et al., 2014). In the healthy stomach, slow waves normally originate from a single pacemaker located in the proximal stomach along the greater-curvature, rapidly establishing ring wavefronts that propagate antegrade toward the distal stomach, terminating at the pylorus (O'Grady et al., 2010a). A number of mathematical models have been developed to study both normal slow wave activity, and a wide range of slow wave abnormalities observed in functional disorders and animal models (Lammers et al., 2012, 2015; O'Grady et al., 2012a; Angeli et al., 2015). This review presents the current state of mathematical modeling of these slow wave abnormalities, and also discusses the methods for validating these models.

### Terminology

Several terminologies have been applied to describe abnormal GI slow wave activities. While some of these terms have been adopted from cardiac electrophysiology, where they have been well-defined, it is worthwhile to also review these terms in the context of their usage in the GI field. Earlier cardiac electrophysiologists pointed out the granular difference between arrhythmias, i.e., absence of rhythm, and dysrhythmias, i.e., abnormalities of rhythm (Trommer, 1982), but usages in practice over the years have converged the two terms to become synonyms (Marriott, 1984). Conventionally in the GI field, dysrhythmias, and arrhythmias have also been used inter-changeably to represent any abnormal slow waves (Lammers, 2013; O'Grady et al., 2014). Nelsen et al., used both arrhythmias and dysrhythmias to describe abnormal slow waves as early as 1968 (Nelsen and Kohatsu, 1968), while other investigators have maintained both usages to describe abnormal slow wave events (Code and Marlett, 1974).

**Figure 1:**
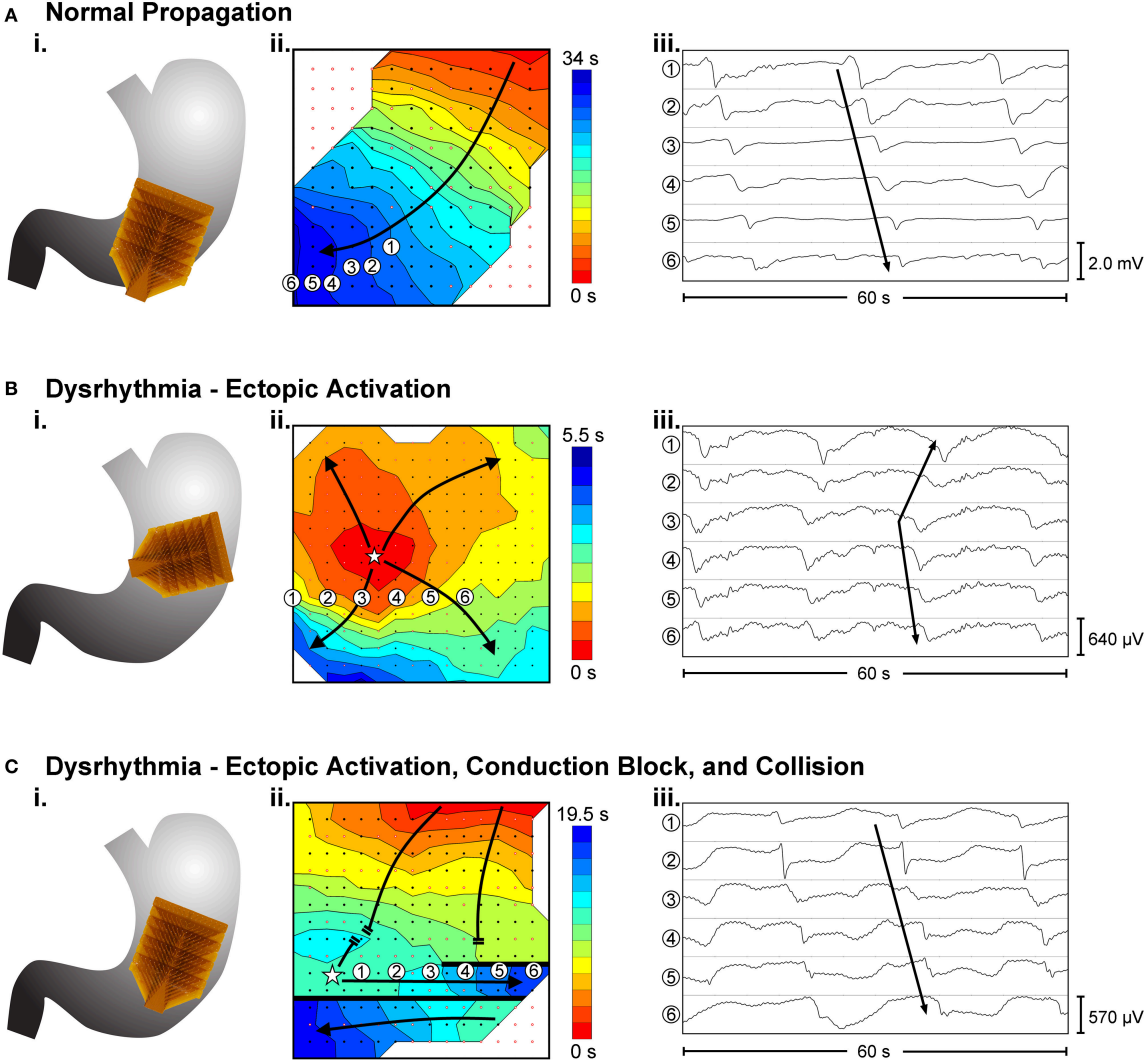
Examples of high-resolution mapping of *in-viv*o gastric slow waves. (i) An array of 16 × 16 electrodes (brown patch) were placed on the serosal surface of the stomach. (ii) Activation times of slow waves were identified and reconstructed into activation maps with red representing early activation and blue presenting late activation. (iii) Example slow wave recordings from six electrodes are shown in each case. **(A)** Normal antegrade propagation pattern of gastric slow wave activation (Angeli et al., 2015). **(B)** An ectopic activation (star) in the proximal stomach. **(C)** Simultaneous ectopic activation, conduction block and collision of slow waves in the gastric corpus. Adapted from (Angeli et al., 2015).

Dysrhythmias can either be defined in terms of frequency, or in terms of spatial pattern. In the stomach, frequency abnormalities have typically been defined as either quiescence, bradygastria, or tachygastria (Code and Marlett, 1974; Stern et al., 1987); while spatial abnormalities have been subjected to a growing list of classifications by various researchers and in various animal models (Table 1). Furthermore, following cardiac conventions, spatial dysrhythmias could also be defined as either abnormal initiation, e.g., ectopic pacemaker, or abnormal conduction, e.g., retrograde propagation. It is worth noting that each of these terms typically describes a single aspect of a dysrhythmia, but in practice dysrhythmic episodes may not occur in isolation. For example, an ectopic initiation event could occur simultaneously with conduction block and collision (Figure 1C), and a conduction block could lead to an aberrant focus of initiation, or a re-entrant conduction abnormality could give rise to secondary conduction abnormalities (O'Grady et al., 2014).

**Table 1:** Definitions of gastric dysrhythmias. Spatial information was obtained from HR mapping taken directly from the gastric serosal surface.

**Terms**	**Definitions**
Arrhythmias/Dysrhythmias (temporal or spatio-temporal)	Abnormal slow wave frequency and/or propagation. Normal human gastric frequency range is typically defined as 2–4 cycle per minute (cpm) (Figure 1) (Owyang and Hasler, 2002).
Quiescent (temporal)	No slow waves (Angeli et al., 2015).
Bradygastria (temporal)	<2 cpm (Parkman et al., 2003; Lim et al., 2012).
Tachygastria (temporal)	> 4 cpm (Parkman et al., 2003; Lim et al., 2012).
Uncoupling (spatio-temporal)	Loss of entrainment leading to two distinct slow wave frequencies in adjacent tissues (Somarajan et al., 2015; Wei et al., 2017).
Anatomical re-entry (spatial)	Self-perpetuating propagation around the circumference of the gut lumen (Angeli et al., 2013b; Du et al., 2017).
Functional re-entry/rotor (spatial)	A rotating wavefront propagating in a single-direction around in a circuit around a central “core” region that acts as a functional conduction block (Lammers et al., 2008b; Angeli et al., 2013b, 2015).
Figure-of-eight/double rotor (spatial)	A single common wavefront that breaks into two rotors propagating in opposite directions (clockwise and anticlockwise) around a core, forming a repeated “figure-of-eight” continuous pattern of activations (Angeli et al., 2013b; Du et al., 2016a).
Conduction block (spatial)	Either a partial or complete block to propagation of normal slow waves (Lammers et al., 2012; O'Grady et al., 2012a; Angeli et al., 2013b).
Retrograde propagation (spatial)	A slow wave event propagating abnormally in the orad direction (O'Grady et al., 2012a; Angeli et al., 2015).
Ectopic activation (spatial)	An ectopic activation was defined as an aberrant initiation of slow waves from a location other than the natural pacemaker (proximal greater curvature) (Lammers et al., 2008b; O'Grady et al., 2012a).
Wave Collision (spatial)	Meeting and termination of two independent wavefronts propagating in opposite directions, e.g., retrograde and antegrade or circumferentially (Lammers et al., 2012, 2015; O'Grady et al., 2012a; Angeli et al., 2015).
Merging wavefronts (spatial)	Joining of two independent wavefronts propagating in the same direction (Angeli et al., 2013b).

*Temporal information was obtained from HR mapping, low resolution serosal recording, and non-invasive recording techniques such EGG and MGG*.

### Dysrhythmias and GI functional disorders

Several GI functional disorders have been associated with slow wave dysrhythmias (O'Grady et al., 2014), as detailed in Table 2, with examples of the types of dysrhythmias that have been reported with each disorder. The majority of previous studies of slow wave activity have utilized low-resolution recording methods, where a sparse number of electrodes were placed on the body-surface (electrogastrography/EGG) or directly on the serosal or mucosal surfaces of the GI organs (Familoni et al., 1987; Lin et al., 1998; Lin and Chen, 2001; Coleski and Hasler, 2009). These studies established foundational discoveries of normal and dysrhythmic slow wave activation, but analysis was largely restricted to the frequency domain, although propagation analysis was also attempted (Simonian et al., 2004; Coleski and Hasler, 2009). The results of these EGG and low-resolution electrode studies thereby focused primarily on ectopic activation as a mechanism of slow wave dysrhythmias (Lin et al., 1998; Lin and Chen, 2001; Simonian et al., 2004).

**Table 2:** Examples of GI functional disorders associated with slow wave dysrhythmias.

**Disorders**	**Recording method(s)**	**Slow wave dysrhythmias reported**
Chronic unexplained nausea and vomiting (Angeli et al., 2015)	HR mapping	Spatiotemporal dysrhythmias occurring across all frequency bands.
Surgical manipulations (Kelly and Code, 1971; Schaap et al., 1990; Du et al., 2015a; Berry et al., 2017)	Low resolution recording, HR mapping	Uncoupling of slow waves across surgical GI bisections; Excisions/incisions led to emergence of rapid circumferential propagation; Frequency abnormalities after gastric resections and anastomoses.
Diabetic dysfunction (intestine) (Lammers et al., 2012, 2015)	HR mapping	Re-entry, ectopic activation with collisions (rodent data).
Mesenteric ischemia (Lammers et al., 1997; Somarajan et al., 2015)	HR mapping, MGG	Uncoupling, with significant decrease in postprandial intestinal slow wave frequency.
Gastroparesis (Lin et al., 1998; O'Grady et al., 2012a)	HR mapping, EGG	Spatiotemporal dysrhythmias occurring across all frequency bands.
Gastroesophageal reflux disease (Leahy et al., 2001; Chen et al., 2004)	EGG	Unstable slow waves, with increased tachygastria in patients with regurgitation.
Systemic sclerosis (McNearney et al., 2009)	EGG	Bradygastria correlated with nausea.
Hyperglycaemia (Hasler et al., 1995; Gonlachanvit et al., 2003; Lien et al., 2003)	EGG	Tachygastria following dextrose infusion.
Chronic intestinal pseudo-obstruction (Debinski et al., 1996)	EGG	Tachygastria, irregular activities, mixture of bradygastria and tachygastria.
Motion sickness (Kim et al., 1997)	EGG	Increase in tachygastria due to vection.
Hyperemesis gravidarum (Koch et al., 1990)	EGG	Mainly tachygastria, with some bradygastria and flat-line activities.
Functional dyspepsia (Pfaffenbach et al., 1997; Leahy et al., 1999; Lin and Chen, 2001; Simonian et al., 2004)	EGG	Increased episodes of tachygastria compared to patients with normal gastric emptying.

More recently, high-resolution GI mapping has emerged as a key methodological advance, where spatially-dense arrays of hundreds of electrodes are placed directly on the GI organ, enabling analysis of slow wave propagation in spatiotemporal detail (Lammers et al., 2008a; Du et al., 2009a). Recent high-resolution mapping studies in patients with diabetic and idiopathic gastroparesis, and chronic unexplained nausea and vomiting, have now demonstrated that spatially-complex dysrhythmias are prevalent in these functional GI disorders (O'Grady et al., 2012a; Angeli et al., 2015). Importantly, many of these spatial dysrhythmias (Table 1) are now known to occur within the normal slow wave frequency range, such that they may likely have gone undiagnosed with previous frequency-reliant methods. Furthermore, it has also been reported that the slow wave dysrhythmias were correlated with loss of the interstitials cells of Cajal (ICC) (O'Grady et al., 2012a; Angeli et al., 2015), which could result in ICC network remodeling (Ordog et al., 2000).

## Current state of GI electrophysiological modeling

Mathematical modeling uses equations to formulate relationships between parameters of a biophysical process. Additional analyses can then be performed on the equations to infer the causal relationship between the parameters over a large parameter space *in silico*, complementary to experimental data, which are needed to validate the model in some capacity. It is worth noting that although a goal is to develop models capable of predictive simulations of physiological functions, no model in its current state can realistically accomplish this ambitious aim in a way that mimics real biological events. Instead, mathematical models are developed to represent electrophysiological mechanisms at discrete biophysical scales as simply and accurately as possible, with input from experimental data at each scale, and attempts are then made to link mechanisms together across these scales.

One particularly significant approach of mathematical modeling is thus to develop a system of equations by following the hierarchy of biological systems across multiple spatiotemporal scales. Such an approach is also called multi-scale modeling, which has been increasingly applied with great effect to study normal and dysrhythmic GI slow waves and dysmotility (Cheng et al., 2013; Du et al., 2013, 2016b). Generally, in a multi-scale model of dysrhythmias the lowest scale relates to the kinetics of the ion channels, which are usually collected into individual ion conductances, with Hill-type activation and inactivation parameters fitted to experimental data (Lees-Green et al., 2011). The dependence of an ion conductance on membrane potential can be modeled using a Hodgkin & Huxley approach, and applied over multiple types of conductance to model the change in slow waves. It is worth noting that the conductance modeled presents an averaged activity of all the same type of conductance in the cell, rather than individual ion channels. As our knowledge of the subcellular processes becomes increasingly clear, more sophisticated techniques such as stochastic modeling and subcellular domain diffusion have also been incorporated to cell models (Lees-Green et al., 2011).

Tissue models require connecting multiple cell models together in a continuum, i.e., spatially averaged, sense has typically been achieved using a reaction-diffusion technique, either in the form of monodomain, bidomain, or tridomain (extended bidomain) equations (Equations 1–6) (Buist and Poh, 2010; Corrias et al., 2012; Du et al., 2013, 2016b; Sathar et al., 2015). The number of domains typically represents the predominant avenues through which the cells are coupled to each other. In the monodomain representation, ICC are assumed to be predominantly coupled via the intracellular domain, whereas in the bidomain representation, the current flow through the extracellular space is also modeled. In contrast to cardiac applications of the tridomain model, which is used to model an additional phase due to non-excitable elements such as fibroblasts (Sachse et al., 2009), the more recently proposed tridomain model represents the coupling between the intracellular spaces of ICC and smooth muscle cells (SMC) and a shared extracellular space (Corrias et al., 2012; Sathar et al., 2015). In each of these cases, under certain assumptions, the higher-order domain models could be reduced to the monodomain model, and extracellular potentials could then be estimated based on the membrane potentials.

Monodomain:

(1) $$\begin{array}{r} \nabla \cdot \left(\sigma \nabla V_{m}\right)=A_{m}\left(C_{m}\frac{\partial V_{m}}{\partial_{t}}+I_{ion}\right) \end{array}$$

Bidomain:

(2) $$\begin{array}{r} \nabla \cdot \left(\left(\sigma_{i}+\sigma_{e}\;\right)\nabla \phi_{e}\;\right)=-\nabla \cdot \left(\sigma_{i}\nabla V_{\text{m}}\right) \end{array}$$

(3) $$\begin{array}{r} \nabla \cdot \left(\sigma_{i}\nabla V_{\text{m}}\right)+\nabla \cdot \left(\sigma_{i}\nabla \phi_{e}\right)=A_{m}\left(C_{m}\frac{\partial V_{m}}{\partial t}+I_{ion}\right) \end{array}$$

Tridomain:

(4) $$\begin{array}{rcl} \nabla \cdot \left(\sigma_{i}^{\left(1\right)}\nabla V_{m}^{\left(1\right)}+\phi_{e}\right) & = & A_{1}\left(C_{m}^{1}\left(\frac{\partial \phi_{i}^{\left(1\right)}}{\partial t}+\frac{\partial \phi_{c}}{\partial t}\right)+I_{ion}^{\left(1\right)}\right)\\ +\;A_{gap}I_{gap} \end{array}$$

(5) $$\begin{array}{rcl} \nabla \cdot \left(\sigma_{i}^{\left(2\right)}\nabla V_{m}^{\left(2\right)}+\phi_{e}\right) & = & A_{2}\left(C_{m}^{2}\left(\frac{\partial \phi_{i}^{\left(2\right)}}{\partial t}+\frac{\partial \phi_{c}}{\partial t}\right)+I_{ion}^{\left(2\right)}\right)\\ -\;A_{gap}I_{gap} \end{array}$$

(6) $$\begin{array}{rcl} \nabla \cdot \left(\sigma_{e}+\sigma_{i}^{\left(1\right)}+\sigma_{i}^{\left(2\right)}\right)\nabla \phi_{e} & = & -\nabla \cdot \left(\sigma_{i}^{\left(1\right)}\nabla V_{m}^{\left(1\right)}\right)\\ -\;\nabla \cdot \left(\sigma_{i}^{\left(2\right)}\nabla V_{m}^{\left(2\right)}\right) \end{array}$$

where σ represents the tissue conductivity, which could be further characterized as intracellular conductivity (σ_*i*_; [S m^−1^]) or extracellular conductivity (σ_*e*_; [S m^−1^]). Membrane potential (V_m_; [V]) can also be expressed as the difference between intracellular potential and extracellular potential, i.e., ϕ_*i*_ − ϕ_*e*_. The total current is denoted by I_ion_ [A]. A_m_ [m^−1^] denotes the surface-to-volume ratio and C_m_ [F m^−2^] denotes the membrane capacitance. In the case of the tridomain equations, conductivities, surface-to-volume ratio, membrane capacitance and intracellular potentials are further distinguished between two cell types, with an added coupling current (I_couple_) between the two cell types. One of the intracellular domains (Equation 4) represents the cytoplasm of ICC, while the other intracellular domain (Equation 5) represents the cytoplasm of SMC. The two intracellular domains share a common extracellular domain (Equation 6).

The choice of the governing equations in the continuum model typically depends on the specific applications. For example, in the absence of detailed measurements of conductivity parameters, applications involving entrainment modeling would typically only require the monodomain model (Du et al., 2015b, 2017), whereas simulations involving an extracellular stimulus would typically require a bidomain/tridomain approach (Sathar et al., 2015). On the other hand, the higher-domain approach would require more computational power due to the increased complexity of formulation. Though direct comparisons between computational times of GI models are difficult to ascertain with varying parameters and numerical solvers involved in the process, on average the computational time of cardiac bidomain models can be an order of magnitude longer than monodmain models (Plank et al., 2009).

Relating the slow wave activity at the whole-organ level to specific individual cells is a challenging, if not impossible, aspect to resolve. Conceptually, the multi-scale models represent slow waves in a spatially-averaged sense, meaning the “continuum” represents an averaged activity of a group of the same cell types within the immediate vicinity in the underlying tissue (Angeli et al., 2013a). A similar loss of spatial information occurs when whole-organ activity is inferred electromagnetically from the body-surface (Kim et al., 2013; Bradshaw et al., 2016b). In this case, the electrophysiological activation of the organ is approximated using dipole(s), which represent the net activation state of the organ at an instance in time. Despite the loss of specificity in the electrical information when upscaling, the higher-level spatial scales also remain a particularly useful approximation of slow waves in the *in vivo* state. The appropriate scale of models should be considered according to the level of mechanisms and specific research questions of interest.

### Cell models

The two main types of biophysically-based cell models being developed over recent years are SMC and ICC, which have been covered extensively in previous reviews (Lees-Green et al., 2011). In general, ICC models incorporate self-excitatory intracellular calcium-based mechanisms to generate slow waves at a specific intrinsic frequency, whereas SMC models require an input stimulus to drive the membrane potentials. Key conductance and/or intracellular calcium dynamics in these cell models are generally fitted to experimental data and validated by reproducing membrane potentials and response to perturbations such as electrical stimuli and drugs. The number of SMC models is limited, including a gastric SMC model derived from animal experimental data (Corrias and Buist, 2007), and uterine SMC derived from uterine human smooth muscle cells (Atia et al., 2016). A list of biophysically-based SMC models is presented in Table 3.

**Table 3:** A list of biophysically-based smooth muscle cell models.

**Smooth Muscle Models**	**Number of ion conductances**
Human colonic smooth muscle cells (Yeoh et al., 2017)	8
Gastric smooth muscle cells (Corrias and Buist, 2007)	8
Human uterine smooth muscle cells (Atia et al., 2016)	27

**Figure 2:**
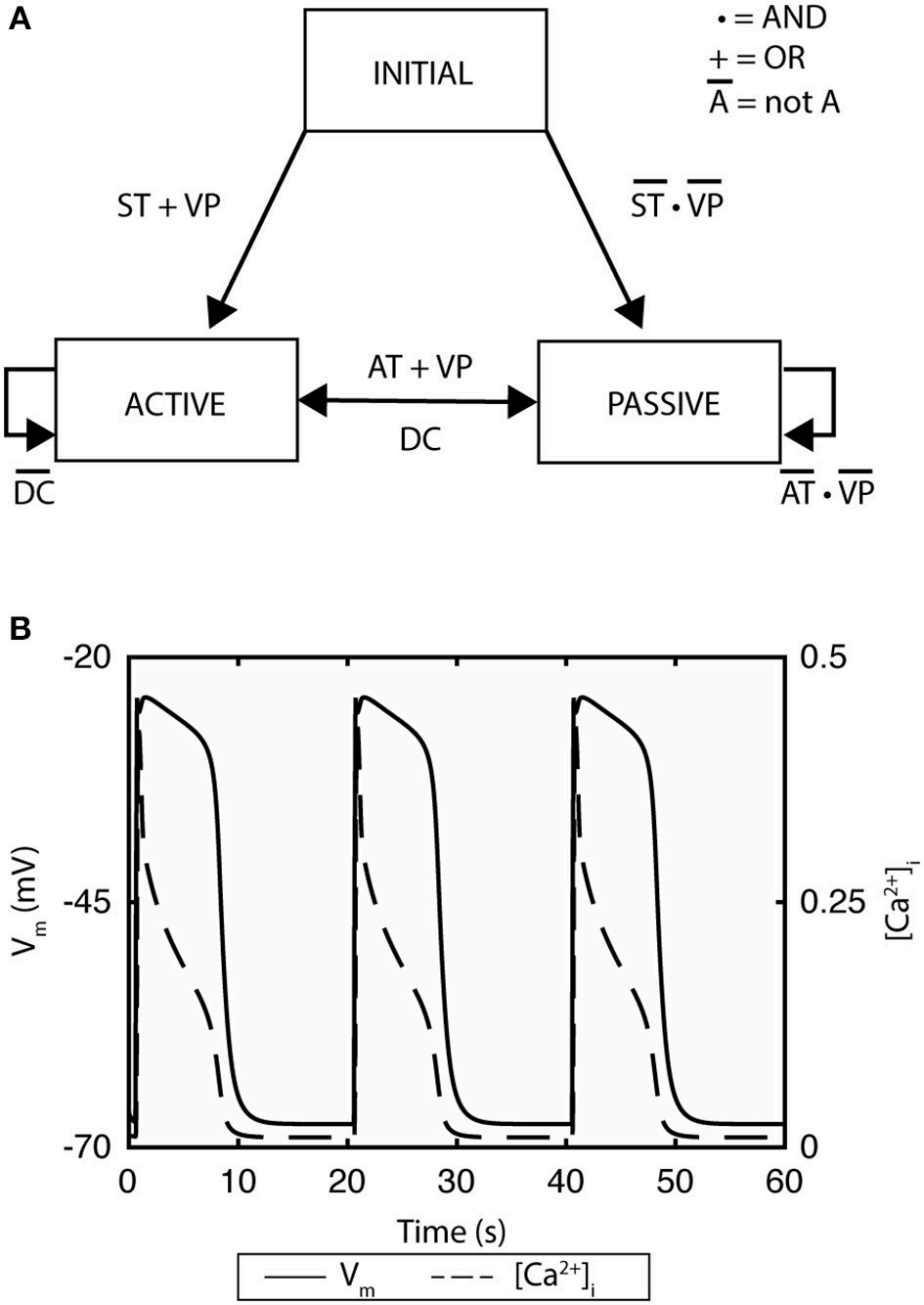
A finite state machine cell model of gastric interstitial cells of Cajal. **(A)** The model consists of an active state and a passive state. ST indicates if time has passed the startTime, which is set as a parameter and which determines initial excitation when there is no threshold voltage. AT indicates if the non-refractory period has been passed and signals transition from passive state to active state. DC identifies if the change in concentration of intracellular Ca^2+^ has returned to quiescent state. VP variable is set to true if there is a voltage which is greater than the threshold of the cell. **(B)** Simulated gastric slow waves and the associated intracellular calcium. Adapted from (Sathar et al., 2014).

A recent example of ICC model development is the finite-state machine based biophysical model by Sathar et al. (2014). In this model, the cellular activity of the model was represented by an active state and passive state, whereby the active state corresponded to the ionic dynamics of the ICC model developed by Corrias and Buist (2008). The transition between the states was modeled using a finite-state machine approach that was dependent on membrane potential, calcium dynamics, and the refractory/non-refractory period parameters defining the intrinsic frequency of the slow wave pacemaker activity (Figure 2A; Sathar et al., 2014). The finite-state machine approach enabled modeling the effects of external current on the cellular activity and has successfully reproduced experimentally observations of gastric pacing. The finite-state machine model is governed by two important rules: (i) if the cellular activity is “active,” it remains active until the calcium dynamics returns to a quiescent state—after which, it changes to a “passive” state; (ii) on the other hand, if the current cellular activity is “passive,” it remains “passive” until the membrane potential exceeds an excitation potential or until the cellular activity has passed the non-refractory period (Figure 2B).

In addition to ICC and SMC, other types of cells have also been proposed to play a role in the electrophysiology and neurotransmission of the GI tract. One such cell is the fibroblast-like cell (FLC), which form discrete networks in the myenteric plexus and are widely distributed within the muscular layer of the colon (Kurahashi et al., 2012). One specific FLC positive for platelet-derived growth factor receptor (PDGFRα^+^) is of particular interest because they are involved in purinergic inhibitory neurotransmissions (Yeoh et al., 2016). Purines released by enteric motor neurons bind to the G-protein coupled receptors in the membrane of the PDGFRα^+^ cells. The binding initiates a series of intracellular processes resulting in the release of Ca^2+^ due to inositol 1,4,5-triphosphate (IP_3_) entering the cytoplasm. The elevation of intracellular Ca^2+^ in turns activates small Ca^2+^-activated K^+^ (SK3) conductance, leading to hyperpolarization of the membrane potential, and potentially also neighboring SMC. Yeoh et al. developed a PDGFRα^+^ cell model by modifying a spatially-independent version of the model proposed by Bennett et al. (2005) and Yeoh et al. (2016), and with their specific description of the SK3 conductance. The authors were able to demonstrate ATP-induced hyperpolarisation and inhibitory effects of MRS2500 and apamin (SK3 channel blocker) (Bennett et al., 2005). The potential applications of this model may be significant in future, as it may be coupled with existing ICC and SMC models to simulate the effects of neural innervation and feedback on slow wave generations and smooth muscle membrane potentials.

Biophysically-based mathematical models of GI cells have been developed at a steady pace, mainly governed by the availability of experimental data and convergence of evidence regarding key electrophysiological mechanisms. However, nearly all GI cell models have been developed as an “after thought” based on published data that were not originally designed with the potential for modeling in mind. The models were generally only able to reproduce the cellular responses over a set of specific interventions that had already been demonstrated experimentally, although some interesting and clinically-relevant applications have already been demonstrated through the predictive modeling of GI channelopathies (Poh et al., 2012). While this is not a unique problem to the GI field, the true predictive power of cell models could be better realized if investigators would consider modeling when developing their experimental design, as has been done in the cardiac field (Maltsev et al., 2017).

### Tissue models

Propagation of slow waves has typically been modeled as a series of coupled phenomenologically-based oscillators, or by employing biophysically-based ICC models. While there are many ways to formulate the specific coupling between ICC models, the ability of an ICC with a higher intrinsic frequency to “entrain” another coupled ICC with lower intrinsic frequency is fundamental (van Helden et al., 2010). The process of entrainment is a key point of distinction between GI and cardiac electrophysiological models. Whereas the cardiac models represent myocytes as single-event active potentials in response to a pacemaker (often modeled as a stimulus), GI models require an interconnected network of pacemakers, where every point in the network is potentially capable of intrinsically generating slow wave activation (Cheng et al., 2013; Du et al., 2013, 2016b). Previous reviews have covered models that have successfully simulated propagation of gastric and intestinal slow waves at the tissue levels (Du et al., 2013, 2016b). This review covers recent work on the simulation of GI slow wave dysrhythmias.

Most recent tissue-level investigations have focused predominantly on the effects of perturbations on the organization of slow waves. Weakly-coupled oscillators have been used to model both intestinal and colonic slow waves (Linkens, 1978; Linkens and Mhone, 1979). This concept was explored further in a recent study to explore the effects of spatial noise in the intrinsic frequency gradient on intestinal slow wave propagation (Parsons and Huizinga, 2016). The study demonstrated that as the coupling (modeled as a gap junction) between the oscillators decreased over a segment of intestine, more slow wave frequency plateaus appeared (Parsons and Huizinga, 2016). One of the conclusions of this study points to the role of a spatial noise on the organization of slow wave propagation and motor patterns in the intestine. As a limitation of the model, the authors pointed out that the intestinal “waxing and waning” phenomenon was not reproduced by the model, but could be explained by a phase-amplitude modulation mechanism (Huizinga et al., 2014). The dynamic interactions between intestinal slow waves in response to a field-stimulus were later quantified using a phase response curve (Parsons and Huizinga, 2017). The weakly-coupled oscillator was extended to a 2D model to reproduce intestinal slow waves in a number of species, as well as the effects of circumferential ICC loss on partitioning of slow waves (Wei et al., 2017).

**Figure 3:**
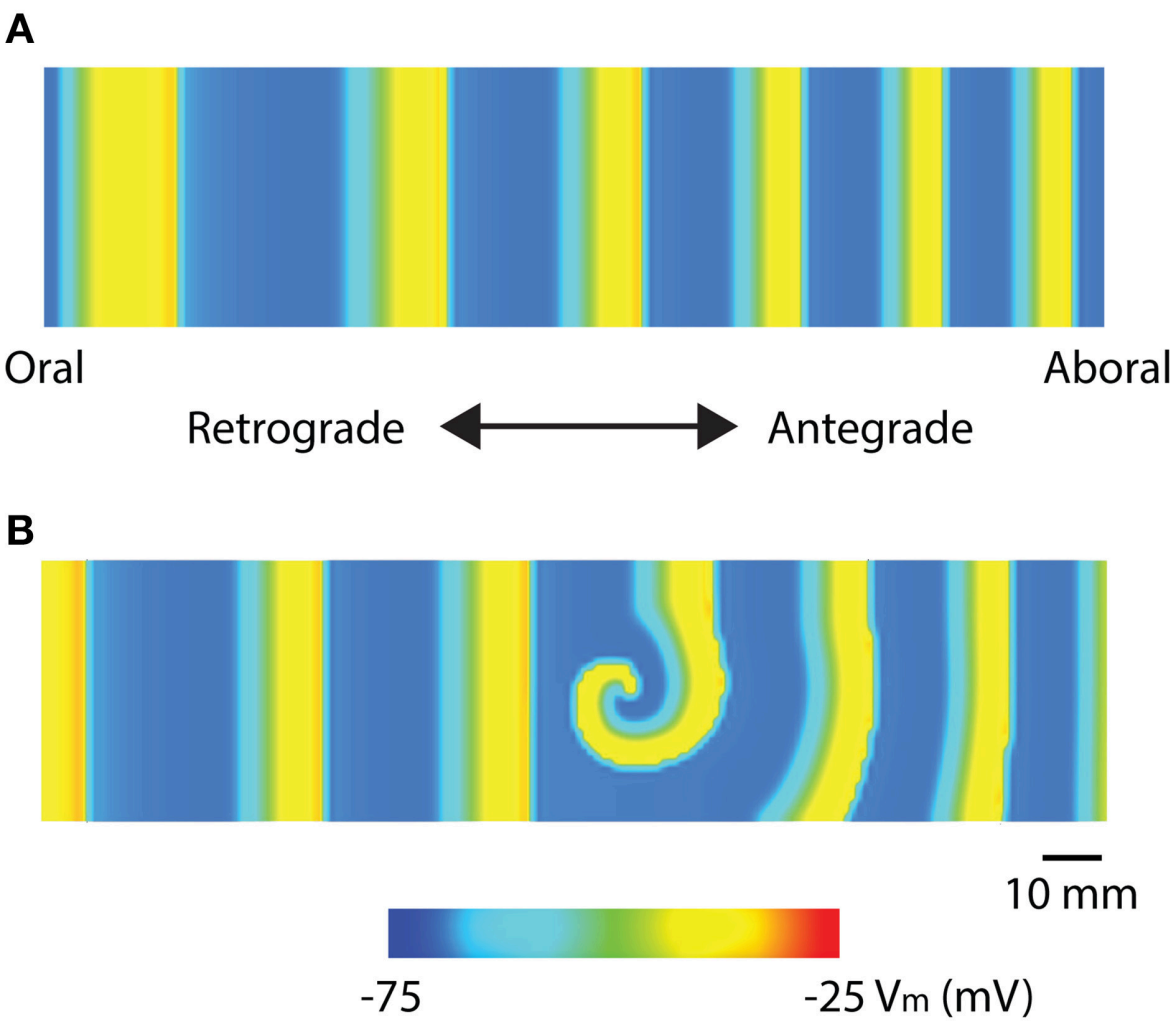
Mathematical models of intestinal slow wave propagation. **(A)** Entrained slow waves over an intrinsic frequency gradient of 17–14.6 cpm (entrained to 17 cpm) were simulated over a 2D model. **(B)** A functional rotor was invoked in the middle of the 2D model using a 30 s long prolonged temporary conduction block. The rotor could be sustained with entrained waves propagating in both antegrade and retrograde directions, with an elevated frequency of 21 cpm. Adapted from (Du et al., 2017).

Recent high-resolution mapping studies have reported re-entry activities in the stomach and intestine (Table 1). Two specific re-entry activities were discovered: *functional re-entry* (Du et al., 2015b), where slow waves propagate in a sustained manner around a core either as in a rotor (Figure 3), or figure-of-eight pattern; and *anatomical re-entry*, where slow wave propagation occurs in continuity around the lumen of the intestine (Angeli et al., 2013b). These re-entry activities were recently modeled in two-dimensional models using a simplified ICC model and the monodomain equation, using a stimulus-driven protocol to invoke the re-entry (Du et al., 2015b, 2017). In order to invoke a rotor in a short segment intestinal tissue, single-pulse stimulus (100 μA mm^−2^; 100 ms) was used to initiate the re-entry next to a temporary conduction block prescribed by the refractory period in the middle of the model (Plank et al., 2009). In particular, it was demonstrated that there was a relatively narrow parameter space in terms of the timing relative to the refractory tail of the previous wavefronts, during which a sustained functional re-entry could be invoked; otherwise, the prescribed intrinsic frequency gradient in the models was essentially able to correct the wavefront over time (Du et al., 2015b, 2017). Anatomical re-entry around the circumference of a 3D lumen was also demonstrated to occur under a similar set of constraints to the functional re-entry, with the added consideration of the geometry of the lumen (Du et al., 2017). One of the features of a sustained re-entry is the elevated frequency associated with the entrainment of slow waves in the core, as demonstrated experimentally (Lammers et al., 2008b). This elevated frequency serves to sustain the re-entry and can lead to entrainment of a substantial section of adjacent intestine. A secondary observation was a frequency-conduction restitution type of relationship where the elevated frequency induces a local reduction in conduction velocity of slow waves (Du et al., 2015b, 2017). Other investigators have modeled the effects of temperature on intestinal slow waves and the formation of re-entry activities (Gizzi et al., 2010).

A bi-directionally coupled model of ICC networks, achieved through coupling of myenteric and intramuscular ICC populations, has been proposed as a homeostatic mechanism in the gut (O'Grady et al., 2012b). Gastric slow waves demonstrate a high degree of anisotropy, whereby slow waves propagate 2.5 times faster in the circumferential axis than the longitudinal axis (O'Grady et al., 2012b; Du et al., 2016a). Similar anisotropy also exists in the small intestine of some species, albeit to a much lesser extent (Angeli et al., 2013b). During normal gastric slow wave propagation, this anisotropy is only observed in the region of the normal pacemaker; thereafter, ring wavefronts are rapidly formed that propagate solely in the longitudinal axis without the need for circumferential spread (O'Grady et al., 2010a). However, during dysrhythmias, the rapid circumferential propagation is routinely observed to emerge in regions adjacent to dysrhythmic sources, due to either aberrant foci of initiation or conduction abnormalities interrupting the normal longitudinal pattern (O'Grady et al., 2012b; Cheng et al., 2013).

The impact of conduction anisotropy was further explored in a simulation where a small full-thickness segment of gastric tissue was excised as part of a biopsy (Du et al., 2015a). Gastric slow wave propagation around this type of excisional biopsy was also mapped experimentally from human subjects. The results demonstrated that area of rapid circumferential conduction was dependent on the orientation of the excision. At all orientations, the slow waves began to normalize in the longitudinal direction at a distance distal to the stapled wound within the field of mapping (60 × 60 mm^2^). It is notable that there is no standard guideline on the dimensions and orientations of gastric excisional procedures in practice (Du et al., 2015a), and these findings could theoretically provide novel insights to minimize post-operative motility changes associated with gastric incisions in future.

In contrast to cell modeling work, recent GI tissue models were generally designed with specific experimental protocols to explore key physiological questions, and/or to provide predictions of potential protocols to invoke sustained dysrhythmia behaviors. Particular efforts have been invested by multiple groups to investigate the organization of intestinal slow waves over extended tissue scales and the formation of re-entry in the stomach and intestine (Gizzi et al., 2010; Du et al., 2015b, 2017; Parsons and Huizinga, 2016). Experimentally, one of the next steps is to attempt to invoke sustained periods of re-entry to thoroughly explore the conditions under which these dysrhythmias occur and possible ways to eliminate them. The proposed relationship between the timing of stimulus and the refractory tail of the preceding wave in invoking re-entry already suggests the potential requirement for monitoring slow wave propagation in real-time experimentally (Bull et al., 2011). Another important avenue of tissue modeling studies has been to explore the structure-function relationship between the ICC network, slow wave propagation and other GI cells, such as the FLC. One potential approach is to reconstruct the structural distributions of these cells from immunofluorescence images and develop a continuum framework in which the interactions between multiple cell types could be taken into account. The structural networks and cellular functions could then be perturbed to explore the effects of ion chancel inhibition and/or cell loss on GI slow waves. Effects of non-electrically active tissues could be modeled by detailed description of the extracellular tissue conductivities in the higher domain models.

### Organ models

The techniques of simulating slow waves over the whole-organ are similar to the tissue models, with a few added considerations (Du et al., 2013, 2016b). First, gastric slow wave propagation has been shown to exhibit significant inter-regional variations within the stomach (Kelly and Code, 1971; Lammers and Stephen, 2008; Egbuji et al., 2010; O'Grady et al., 2010a). *In vivo* high-resolution mapping data have demonstrated that the fundus is generally quiescent in large monogastric species, and a pacemaker region exists in the proximal corpus along the greater curvature, from which gastric slow waves rapidly form ring wavefronts that propagate distally toward the antrum (Lammers and Stephen, 2008; Egbuji et al., 2010; O'Grady et al., 2010a). In humans, a marked acceleration of slow wave activation is then seen in the region of the terminal antrum, just proximal to the pylorus, which constitutes the basis of the terminal antral contraction (Berry et al., 2016). Second, a resting membrane potential gradient due to the action of ICC-derived carbon monoxide as a hyperpolarizing agent exists from the proximal to the distal stomach, as well as across the gastric and small intestinal wall (Farrugia et al., 2003). In the colon, the direction of the resting membrane potential gradient across the colonic wall is reversed compared to the stomach and intestine (Szurszewski and Farrugia, 2004). Finally, gastric slow waves entrain to a singular frequency in the healthy stomach, whereas in the intestine slow waves occur at multiple frequencies organized into plateaus along the length of the organ, each governed by an independent pacemaker, effectively forming a step-wise gradient of decreasing frequency along the intestine (Diamant and Bortoff, 1969; Lammers and Stephen, 2008; Angeli et al., 2013c). Previous reviews have covered studies that have attempted to unify all three aspects in a single model by prescribing parameters in the cell model to reproduce the intrinsic frequency, resting membrane potential gradients, and the conduction velocities in each region of the stomach (Du et al., 2010a, 2013). Recent modeling studies have now expanded on the whole-organ model to investigate the mechanisms of slow wave recovery and gastric dysrhythmias (Calder et al., 2016; Paskaranandavadivel et al., 2017).

**Figure 4:**
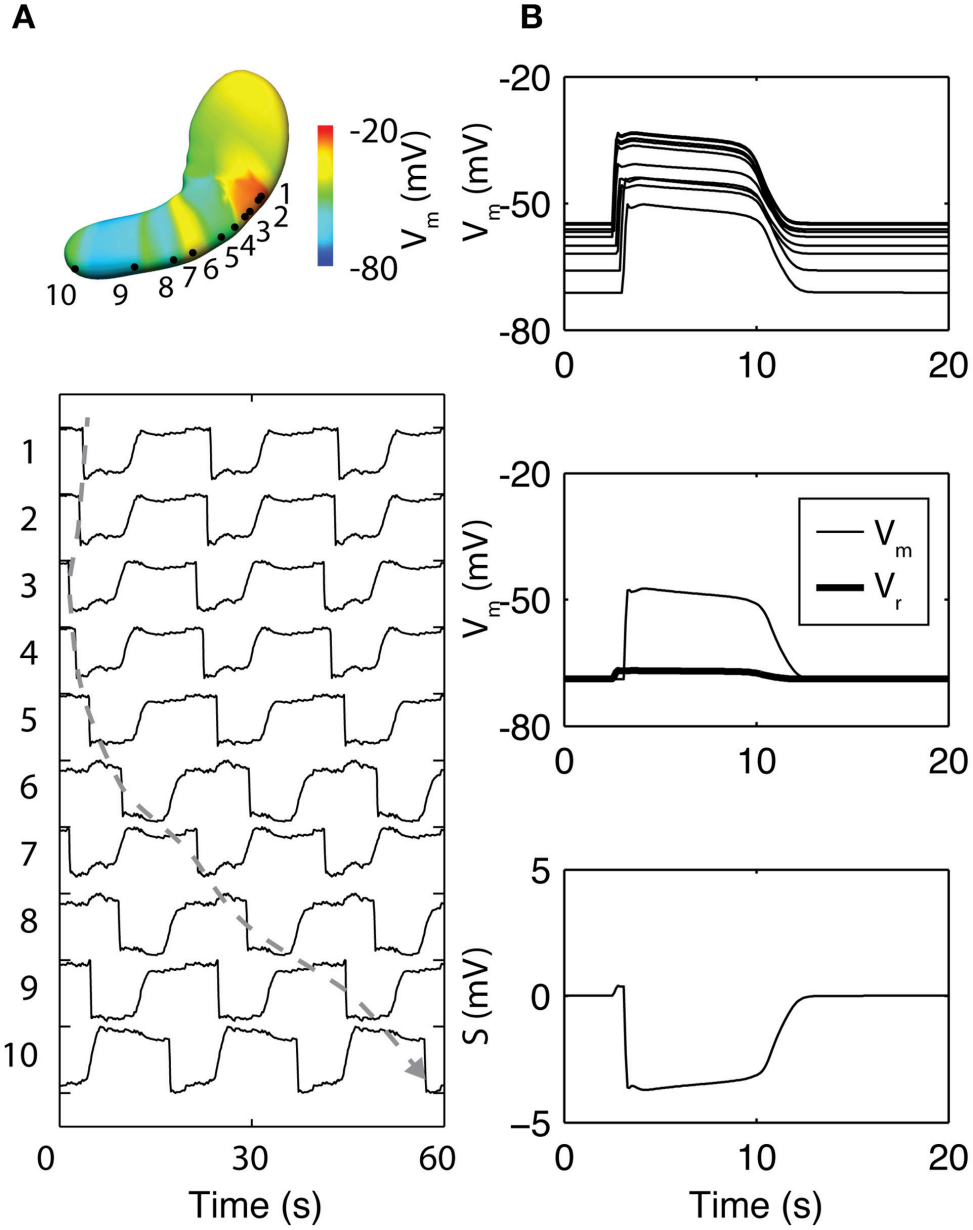
Simulation of whole-organ gastric slow waves. **(A)** Gastric slow waves originate from a pacemaker region along the greater curvature in the proximal stomach. Simultaneous and multiple wavefronts occur in the stomach, with each propagating wavefront taking up to 60 s to reach to the terminal antrum. **(B)** The existence of resting membrane potential gradient in the stomach plays a key role to the recovery component in the extracellular signals, when calculated as a difference between membrane potential (V_m_) and a spatially invariant term (V_r_). Adapted from (Paskaranandavadivel et al., 2017).

In a notable recent study, the basis of the slow wave refractory/recovery was explored by sampling simulated slow waves from 96 solution points sampled over a whole-organ model (Paskaranandavadivel et al., 2017). The unipolar potential recorded in standard extracellular mapping techniques was simulated as a combination of a local component, i.e., membrane potential, and a spatially-independent component based on a scaled average of the residuals between the local component and the extracellular potentials from the whole-organ (Figure 4; Paskaranandavadivel et al., 2017). The simulations demonstrated that the resting membrane potential gradient may be a significant contributor to the recovery phase of the gastric slow wave extracellular potential. Specifically focusing on the spatially-independent component (Figure 4B), the gradient of resting membrane potentials had the effect of “averaging out” the defined profile of the spatially-independent component, as also seen in the cardiac simulations (Potse et al., 2009). The result was a pronounced recovery phase in the extracellular potential (Figure 4B).

**Figure 5:**
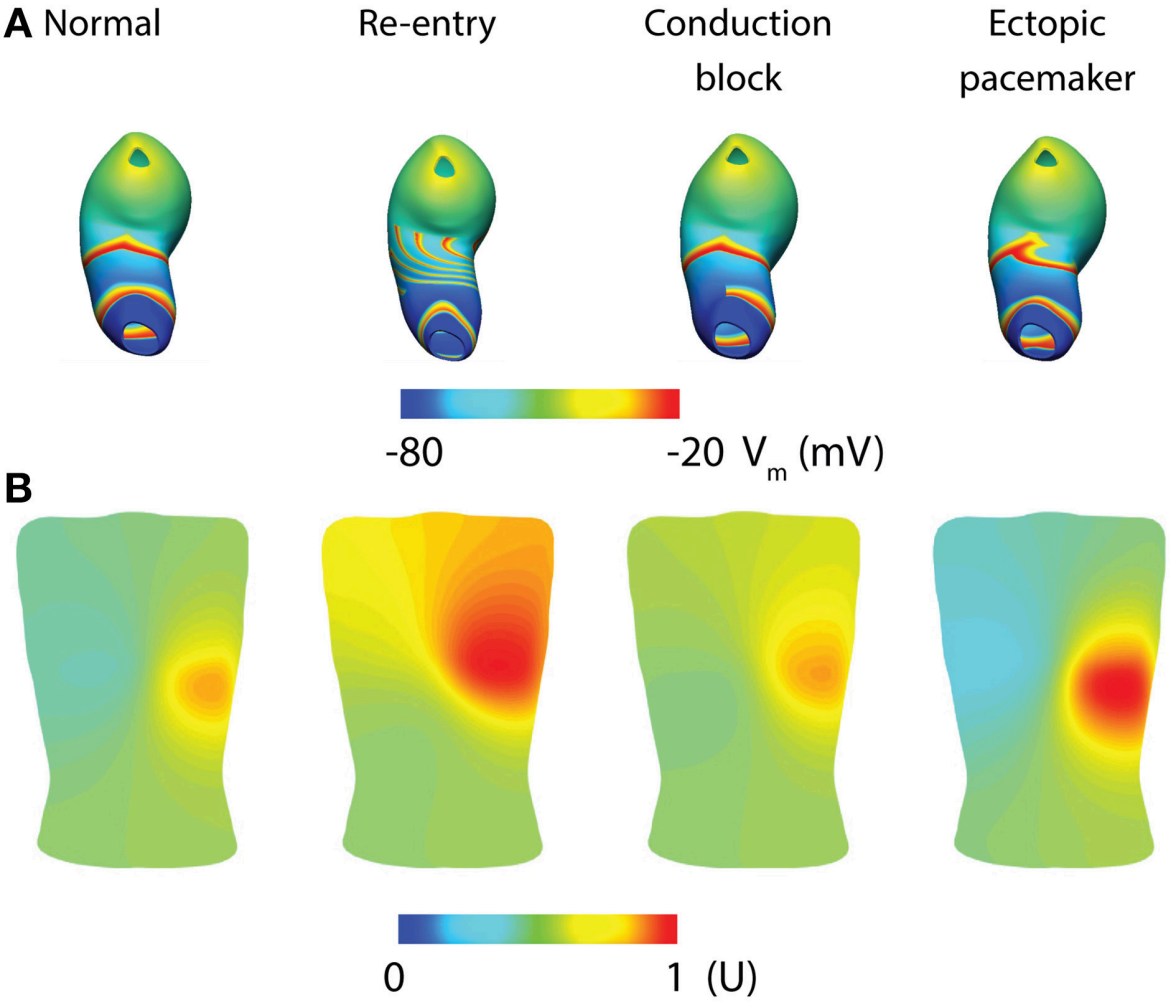
Whole-organ gastric slow wave dysrhythmias and electrogastrography (EGG) simulations (Calder et al., 2016). **(A)** Four instance of gastric slow wave activation (normal, re-entry, conduction block in the antrum and ectopic pacemaker in the proximal stomach). **(B)** Corresponding EGG simulations are calculated using a forward approach over an anatomically realistic torso, with the EGG potentials normalized (U).

Whole-organ gastric dysrhythmias were simulated by adding a stimulus driven protocol and/or conduction block based on the tissue models (Calder et al., 2016). Three dysrhythmias have been reproduced to date: re-entry, conduction block and ectopic pacemaker (Figure 5; see Table 1 for definitions). Gastric re-entry was successfully invoked in the corpus where the distance between the refractory-depolarization of two subsequent waves is the longest, allowing more time for re-entry to develop. Both conduction blocks and ectopic pacemakers (of equal frequency to the entrainment frequency) were prescribed to the antrum, where the two dysrhythmias have been most commonly identified. The potential applications of these whole-organ gastric dysrhythmia models include investigations of forward and inverse modeling techniques, relating the effects of dysrhythmias with motility through computational fluid dynamic studies, and in identifying enhanced methods of gastric pacing (Berry et al., 2016).

Whole-organ modeling studies of slow waves have mainly focused on the stomach, where both *in vivo* and *in vitro* data are most abundant. With the development of a foundational gastric model, recent investigations have utilized this model to investigate detailed mechanisms for physiological recordings, as well as simulations of dynamic dysrhythmias over the entire organ. Similar intestinal models will likely be developed in idealized geometry segments, as the overall intestinal length of multiple meters poses a significant computational challenge to the current models that rely on a high spatial resolution of solution points (<0.3 mm). Furthermore, there is also uncertainty in whether some of the “abnormal” intestinal slow waves are truly dysrhythmic, or rather occur as stochastic behaviors as part of the natural pacemaking and/or dynamic interactions between intestinal slow wave plateaus (Parsons and Huizinga, 2016, 2017).

### Non-invasive recordings

One of the ultimate aims of GI slow wave recordings is the ability to more reliably interpret slow wave activities non-invasively from the body surface. For gastric slow waves, the interpretation has principally been done through cutaneous recordings known as electrogastrography (EGG) (Bortolotti, 1998). It is also possible to detect the magnetic fields associated with the generation of slow waves with a superconducting quantum interference device (SQUID) using a technique called magnetogastrography (MGG) (Somarajan et al., 2015; Bradshaw et al., 2016b). Modeling investigations on both techniques have been ongoing as our understanding of the detailed activation sequence of gastric slow waves has improved. In particular, investigators are increasingly focusing on multi-channel/high-resolution EGG for detecting the underlying gastric slow wave propagation (Gharibans et al., 2016).

Given the relatively low signal-to-noise ratio of EGG, and the complexity of the underlying sources, investigators have long struggled with reliably separating the EGG signal component from other sources, e.g., respiration interference and cardiac electrical activity, though signal processing techniques have recently been developed to minimize these contaminants (Komorowski and Tkacz, 2015; Komorowski and Pietraszek, 2016). As a result, the sensitivity and specificity of the EGG has remained suboptimal, limiting clinical uptake. Mathematical modeling offers an attractive platform to assist in further development, because it can provide a “noise free” environment in which simulated EGG can be directly matched with gastric slow waves, provided that the correct slow wave behaviors are reproduced. A recent modeling investigation studied the EGG associated with normal and dysrhythmias gastric slow waves as demonstrated by high-resolution mapping (Calder et al., 2016). The whole-organ simulations were conducted as outlined in the whole-organ section of this review above (Figure 5A). The network activation of the whole-organ slow waves was represented using a dipole, which was placed inside an anatomically-realistic model of the adult torso with 192 cutaneous EGG electrodes (Figure 5B; Calder et al., 2016). EGG was modeled using the boundary-element method with the assumption of homogeneity inside the torso. The main finding was that, in addition to detecting the frequency of slow waves, it is also theoretically possible to detect and reliably distinguish spatial dysrhythmias (re-entry, conduction block, and ectopic pacemaker) using multi-channel (HR) EGG. Alternatively, slow wave activation has also been modeled as a 1D propagation source, and EGG calculated as a weighted summation of slow waves at every instance in time (Gharibans et al., 2016). Crucially, this study also used surface Laplacian and wave estimation techniques to determine the direction and propagation of slow waves from EGG recorded from eight human subjects (Gharibans et al., 2016). The clinical significance of these interesting recent developments will be more clear once the ability to interpret clinical EGG recordings improves.

The relationship between slow wave dysrhythmias and MGG has been validated experimentally with gastric disease models and intestinal ischemia (Bradshaw et al., 2007, 2016a; Somarajan et al., 2015). Investigators have resolved the theoretical relationship of MGG to normal gastric slow wave activation. One recent study compared the surface current density method and a second-order blind source separation method to the theoretical prediction of the source location based on a dipole, and found close agreement with the measurements and the predicted data (Bradshaw et al., 2016b). This study also raised the question of whether gastric slow waves can be well captured using a single dipole, or better represented by multiple dipoles to track the simultaneous propagating wavefronts.

The current progress in non-invasive recording techniques is mirrored with the improved understanding of the underlying gastric slow wave activation. The technical capabilities of inferring data from multiple channels are allowing more quantifiable techniques to be applied to EGG and MGG. Most simulation studies in this field have updated their source to represent a more realistic activation pattern based on experimental data. However, questions will always remain regarding the sensitivity of the EGG and MGG until experimental studies can definitively prove that the EGG and MGG can be related to specific spatial activation states of gastric slow waves, beyond just a frequency correlation, which will require simultaneous HR recordings from the stomach and body-surface. In addition, reliable protocols for invoking specific types of dysrhythmias in a consistent manner are also still needed to determine the relationship between EGG/MGG and dysrhythmias. One potential such method, for example, is through high-energy gastric pacing to create a stable ectopic activation (Du et al., 2009b; O'Grady et al., 2010b; Sathar et al., 2015).

## Simulation environments and standards

As models increase in scale there is an associated increase in the computational cost in order to solve these models. This is especially important in GI modeling because the time-scale is typically orders of magnitudes higher than neural and cardiac simulations (milliseconds to minutes vs. milliseconds). Furthermore, as models gain sophistication it is ever more important to develop a standard by which these models can be encoded and reproduced in a consistent manner, as well as for encapsulation of meta-data. To this end, a number of standards and simulation tools have been developed (Table 4). The list of tools in Table 4 is by no means exhaustive, but is listed to highlight some examples of the available modeling standards and tools. One important reason for adopting similar standards is to allow rapid exchange of information and reproducibility of simulation results.

**Table 4:** Examples of markup standards for encoding models and simulation environments.

**Name**	**Programming language**	**Purpose**
CellML (Lloyd et al., 2004) www.cellml.org	XML	Encoding subcellular and cellular processes
SBML (Chaouiya et al., 2013) http://sbml.org	XML	Systems Biology Markup Language for encoding subcellular and cellular processes
FieldML (Christie et al., 2009) www.fieldml.org	XML	Modeling and interchanging spatially-varying field parameters.
CMISS www.cmiss.org	Fortran	A multi-scale simulation tool.
OpenCMISS (Bradley et al., 2011) http://opencmiss.org/	Fortran	A distributed parallel mathematical modeling environment for multi-scale simulations.
Chaste (Pitt-Francis et al., 2008) http://www.cs.ox.ac.uk/chaste/	C/C++	A distributed parallel mathematical modeling environment for multi-scale simulations.
Continuity http://www.continuity.ucsd.edu	Python	Multi-scale modeling and data analysis

## Final remarks and future direction

Mathematical modeling of GI electrophysiology has seen remarkable progress in recent years. While still lacking the depth of detailed understanding in physiological and pathological mechanisms that exists in the cardiac and neural fields, the recent advance in cellular slow wave mechanisms and the detailed spatiotemporal descriptions of gastric spatial dysrhythmias have enabled a new focus and level of impact for many GI modeling studies. As new knowledge of cellular ion channelopathies and tissue degradation in diseases becomes available (Beyder and Farrugia, 2016), existing models can be updated with improved specificity to understand a particular pathology, infer its influence on the organ through multi-scale modeling, and determine detectability through non-invasive recording methodologies.

As biological modeling is often informed by experimental data, the focus on technical setup in experiments is becoming increasingly important. For example, EGG and MGG at the body surface have different signal characteristics compared to the signals recorded by direct contact on the serosal surface. Therefore, more consideration needs to be given to the choice of amplifiers and signal processing techniques. Inappropriate applications of recording and/or processing techniques could lead to incorrect interpretation of signals (Paskaranandavadivel et al., 2013). Furthermore, there is a danger of relying on recordings at one scale to interpret recordings obtained at a different scale. For example, some investigators have incorrectly discussed how the slow wave signals recorded using extracellular mapping do not appear to have the same rate of change as the intracellular recordings obtained from individual cells (Sanders et al., 2016). This interpretation relies on the false presumption that the extracellular serosal recordings directly accord with the activation of an individual cell, which is not the case in practice (Angeli et al., 2013a; O'Grady et al., 2017).

The role of the structure-function relationship of ICC and smooth muscles in GI dysrhythmias is a critical current question in clinical GI motility. Structural imaging and calcium functional recordings have both pointed to a strong relationship between the spatial distribution of ICC/cellular manipulations and loss of synchronicity of calcium activities and motility (Singh et al., 2014; Malysz et al., 2017), which are associated with slow wave dysrhythmias. At present, imaging and functional recording techniques are applied in isolation. It would be of great value to develop a foundational modeling framework that can integrate the experimental data from different modalities using biophysically-based models. Previous modeling investigations have attempted simulating slow wave propagation over a small-scale ICC network, either obtained from imaging data or using network generation algorithms (Du et al., 2010b; Gao et al., 2015). Validation for these studies was difficult because functional recording of the tissue-specific networks was not available at the time. With the advent of ratiometric Ca^2+^ imaging in live GI tissues (Singh et al., 2014; Malysz et al., 2017), it is now possible to compare the simulated intracellular Ca^2+^ activities that accompany slow wave generation to experimental data under varied physiological conditions.

The capability of recording Ca^2+^ transients at the tissue level could also lead to the development of new electromechanical models (Du et al., 2011; Singh et al., 2014; Malysz et al., 2017). This is critical because motility is a functional consequence of slow wave activation, and ICC have been shown to exhibit significant mechano-sensitivity (Beyder et al., 2010), which has been studied using a mathematical cell model (Poh et al., 2012). Both a passive constitutive model and an active tension model should be developed in order to translate the electromechanical coupling to the tissue level. Experiments based on biaxial stretch of the GI tissues are needed to quantify the parameters for the constitutive models, while a steady-state tension–length–calcium relationship could be adapted to define the active tension model. Perturbations to ion channel and tissue conductances could then be prescribed to the electrometrical models to simulate dysmotility. When translated to the whole-organ level, the simulated deformation of the GI wall could be used as a boundary condition input to computational fluid dynamic simulations of the mixing and breakdown of luminal contents due to different motility patterns (Fullard et al., 2014).

In summary, recent GI mathematical models have increased in sophistication and their ability to simulate key physiological mechanisms. These models have been applied in a predictive manner, across multiple biophysical scales, to begin to address some of the long-standing questions in the GI field, including the mechanisms, significance, and non-invasive diagnostics of gastric dysrhythmias. The progress of models relating to slow wave dysrhythmias is encouraging to date. However, as both experimental and technical capabilities within the field continue to improve, models will be better informed and validated by additional types of data. In turn, mathematical models of GI slow wave activity will continue to provide an ever-expanding platform for an integrative understanding of experimental data across multiple modalities.

## Author contributions

PD: manuscript preparation, study design. SC, TA, GO, NP, SS and LC: manuscript preparation. SS and NP contributed to drafting and preparation of the manuscript.

### Conflict of interest statement

PD, TA, NP, GO, and LC hold intellectual property/patent applications in the field of mapping gastrointestinal electrophysiology. PD, TA, NP, SS, GO, LC are shareholders in FlexiMap. No commercial or industry funding was provided for this work.

## References

[B1] Angeli T. R. Cheng L. K. Du P. Wang T. H. Bernard C. E. Vannucchi M. G.. (2015). Loss of interstitial cells of cajal and patterns of gastric dysrhythmia in patients with chronic unexplained nausea and vomiting. Gastroenterology 149, 56-66 e5. 10.1053/j.gastro.2015.04.003 25863217 PMC4617790

[B2] Angeli T. R. Du P. Paskaranandavadivel N. Janssen P. W. Beyder A. Lentle R. G.. (2013a). The bioelectrical basis and validity of gastrointestinal extracellular slow wave recordings. J. Physiol. 591, 4567–479. 10.1113/jphysiol.2013.254292 23713030 PMC3784199

[B3] Angeli T. R. O'Grady G. Du P. Paskaranandavadivel N. Pullan A. J. Bissett I. P.. (2013c). Circumferential and functional re-entry of *in vivo* slow-wave activity in the porcine small intestine. Neurogastroenterol. Motil. 25, e304–e314. 10.1111/nmo.12085 23489929 PMC3781238

[B4] Angeli T. R. O'Grady G. Paskaranandavadivel N. Erickson J. C. Du P. Pullan A. J.. (2013b). Experimental and automated analysis techniques for high resolution electrical mapping of small intestine slow wave activity. J. Neurogastroenterol. Motil. 19, 179–191. 10.5056/jnm.2013.19.2.179 23667749 PMC3644654

[B5] Atia J. McCloskey C. Shmygol A. S. Rand D. A. H. A. van den Berg Blanks A. M. (2016). Reconstruction of cell surface densities of ion pumps, exchangers, and channels from mRNA expression, conductance kinetics, whole-cell calcium, and current-clamp voltage recordings, with an application to human uterine smooth muscle cells. PLoS Comput. Biol. 12:e1004828. 10.1371/journal.pcbi.1004828 27105427 PMC4841602

[B6] Bennett M. R. Farnell L. Gibson W. G. (2005). A quantitative model of purinergic junctional transmission of calcium waves in astrocyte networks. Biophys. J. 89, 2235–2250. 10.1529/biophysj.105.062968 16055527 PMC1366726

[B7] Berry R. Cheng L. K. Du P. Paskaranandavadivel N. Angeli T. R. Mayne T.. (2017). Patterns of abnormal gastric pacemaking after sleeve gastrectomy defined by laparoscopic high-resolution electrical mapping. Obes. Surg. 27, 1929–1937. 10.1007/s11695-017-2597-6 28213666

[B8] Berry R. Miyagawa T. Paskaranandavadivel N. Du P. Angeli T. R. Trew M. L.. (2016). Functional physiology of the human terminal antrum defined by high-resolution electrical mapping and computational modeling. Am. J. Physiol. Gastrointest. Liver Physiol. 311, G895–G902. 10.1152/ajpgi.00255.2016 27659422 PMC5130547

[B9] Beyder A. Farrugia G. (2016). Ion channelopathies in functional GI disorders. Am. J. Physiol. Gastrointest. Liver Physiol. 311, G581–G586. 10.1152/ajpgi.00237.2016 27514480 PMC5142191

[B10] Beyder A. Rae J. L. Bernard C. Strege P. R. Sachs F. Farrugia G. (2010). Mechanosensitivity of Nav1.5, a voltage-sensitive sodium channel. J. Physiol. 588, 4969–4985. 10.1113/jphysiol.2010.199034 21041530 PMC3036191

[B11] Bortolotti M. (1998). Electrogastrography: a seductive promise, only partially kept. Am. J. Gastroenterol. 93, 1791–1794. 10.1111/j.1572-0241.1998.01791.x 9772032

[B12] Bradley C. Bowery A. Britten R. Budelmann V. Camara O. Christie R.. (2011). OpenCMISS: a multi-physics & multi-scale computational infrastructure for the VPH/Physiome project. Prog. Biophys. Mol. Biol. 107, 32–47. 10.1016/j.pbiomolbio.2011.06.015 21762717

[B13] Bradshaw L. A. Cheng L. K. Chung E. Obioha C. B. Erickson J. C. Gorman B. L.. (2016a). Diabetic gastroparesis alters the biomagnetic signature of the gastric slow wave. Neurogastroenterol. Motil. 28, 837–848. 10.1111/nmo.12780 26839980 PMC4877247

[B14] Bradshaw L. A. Kim J. H. Somarajan S. Richards W. O. Cheng L. K. (2016b). Characterization of electrophysiological propagation by multichannel sensors. IEEE Trans. Biomed. Eng. 63, 1751–1759. 10.1109/TBME.2015.2502065 26595907 PMC4873475

[B15] Bradshaw L. A. Sims J. A. Richards W. O. (2007). Noninvasive assessment of the effects of glucagon on the gastric slow wave. Am. J. Physiol. Gastrointest. Liver Physiol. 293, G1029–G1038. 10.1152/ajpgi.00054.2007 17884978 PMC2726773

[B16] Buist M. L. Poh Y. C. (2010). An extended bidomain framework incorporating multiple cell types. Biophys. J. 99, 13–18. 10.1016/j.bpj.2010.03.054 20655828 PMC2895333

[B17] Bull S. H. O'Grady G. Cheng L. K. Pullan A. J. (2011). A framework for the online analysis of multi-electrode gastric slow wave recordings. Conf. Proc. IEEE Eng. Med. Biol. Soc. 2011, 1741–1744. 10.1109/IEMBS.2011.6090498 22254663 PMC4108589

[B18] Calder S. O'Grady G. Cheng L. Du P. (2016). A theoretical analysis of electrogastrography (EGG) signatures associated with gastric dysrhythmias. IEEE Trans. Biomed. Eng. 64, 1592–1601. 10.1109/TBME.2016.2614277 28113227

[B19] Chaouiya C. Berenguier D. Keating S. M. Naldi A. van Iersel M. P. Rodriguez N.. (2013). SBML qualitative models: a model representation format and infrastructure to foster interactions between qualitative modelling formalisms and tools. BMC Syst. Biol. 7:135. 10.1186/1752-0509-7-135 24321545 PMC3892043

[B20] Chen C. L. Lin H. H. Huang L. C. Huang S. C. Liu T. T. (2004). Electrogastrography differentiates reflux disease with or without dyspeptic symptoms. Dig. Dis. Sci. 49, 715–719. 10.1023/B:DDAS.0000030079.20501.62 15259489

[B21] Cheng L. K. Du P. O'Grady G. (2013). Mapping and modeling gastrointestinal bioelectricity: from engineering bench to bedside. Physiology 28, 310–317. 10.1152/physiol.00022.2013 23997190 PMC3768093

[B22] Christie G. R. Nielsen P. M. Blackett S. A. Bradley C. P. Hunter P. J. (2009). FieldML: concepts and implementation. Philos. Trans. A Math. Phys. Eng. Sci. 367, 1869–1884. 10.1098/rsta.2009.0025 19380316 PMC2665020

[B23] Code C. F. Marlett J. A. (1974). Canine tachygastria. Mayo Clin. Proc. 49, 325–332. 4829263

[B24] Coleski R. Hasler W. L. (2009). Coupling and propagation of normal and dysrhythmic gastric slow waves during acute hyperglycaemia in healthy humans. Neurogastroenterol Motil 21, 492-9, e1–2. 10.1111/j.1365-2982.2008.01235.x 19309443

[B25] Corrias A. Buist M. L. (2007). A quantitative model of gastric smooth muscle cellular activation. Ann. Biomed. Eng. 35, 1595–1607. 10.1007/s10439-007-9324-8 17486452

[B26] Corrias A. Buist M. L. (2008). Quantitative cellular description of gastric slow wave activity. Am. J. Physiol. Gastrointest. Liver Physiol. 294, G989–G995. 10.1152/ajpgi.00528.2007 18276830

[B27] Corrias A. Pathmanathan P. Gavaghan D. J. Buist M. L. (2012). Modelling tissue electrophysiology with multiple cell types: applications of the extended bidomain framework. Integr. Biol. 4, 192–201. 10.1039/c2ib00100d 22222297

[B28] Debinski H. S. Ahmed S. Milla P. J. Kamm M. A. (1996). Electrogastrography in chronic intestinal pseudoobstruction. Dig. Dis. Sci. 41, 1292–1297. 10.1007/BF02088549 8689901

[B29] Diamant N. E. Bortoff A. (1969). Nature of the intestinal slow-wave frequency gradient. Am. J. Physiol. 216, 301–307. 4975008 10.1152/ajplegacy.1969.216.2.301

[B30] Du P. Hameed A. Angeli T. R. Lahr C. Abell T. L. Cheng L. K.. (2015a). The impact of surgical excisions on human gastric slow wave conduction, defined by high-resolution electrical mapping and in silico modeling. Neurogastroenterol. Motil. 27, 1409–1422. 10.1111/nmo.12637 26251163 PMC4598186

[B31] Du P. O'Grady G. Cheng L. K. (2017). A theoretical analysis of anatomical and functional intestinal slow wave re-entry. J. Theor. Biol. 425, 72–79. 10.1016/j.jtbi.2017.04.021 28450068

[B32] Du P. O'Grady G. Cheng L. K. Pullan A. J. (2010a). A multiscale model of the electrophysiological basis of the human electrogastrogram. Biophys. J. 99, 2784–2792. 10.1016/j.bpj.2010.08.067 21044575 PMC2965998

[B33] Du P. O'Grady G. Egbuji J. U. Lammers W. J. Budgett D. Nielsen P.. (2009a). High-resolution mapping of *in vivo* gastrointestinal slow wave activity using flexible printed circuit board electrodes: methodology and validation. Ann. Biomed. Eng. 37, 839–846. 10.1007/s10439-009-9654-9 19224368 PMC4090363

[B34] Du P. O'Grady G. Gao J. Sathar S. Cheng L. K. (2013). Toward the virtual stomach: progress in multiscale modeling of gastric electrophysiology and motility. Wiley Interdiscip. Rev. Syst. Biol. Med. 5, 481–493. 10.1002/wsbm.1218 23463750 PMC3681930

[B35] Du P. O'Grady G. Gibbons S. J. Yassi R. Lees-Green R. Farrugia G.. (2010b). Tissue-specific mathematical models of slow wave entrainment in wild-type and 5-HT(2B) knockout mice with altered interstitial cells of Cajal networks. Biophys. J. 98, 1772–1781. 10.1016/j.bpj.2010.01.009 20441740 PMC2862206

[B36] Du P. O'Grady G. Paskaranandavadivel N. Tang S. J. Abell T. Cheng L. K. (2016a). Simultaneous anterior and posterior serosal mapping of gastric slow-wave dysrhythmias induced by vasopressin. Exp. Physiol. 101, 1206–1217. 10.1113/EP085697 PMC5140776 27265885

[B37] Du P. O'Grady G. Windsor J. A. Cheng L. K. Pullan A. J. (2009b). A tissue framework for simulating the effects of gastric electrical stimulation and *in vivo* validation. IEEE Trans. Biomed. Eng. 56, 2755–2761. 10.1109/TBME.2009.2027690 19643697 PMC4169301

[B38] Du P. Paskaranandavadivel N. Angeli T. R. Cheng L. K. O'Grady G. (2016b). The virtual intestine: *in silico* modeling of small intestinal electrophysiology and motility and the applications. Wiley Interdiscip. Rev. Syst. Biol. Med. 8, 69–85. 10.1002/wsbm.1324 26562482 PMC5097873

[B39] Du P. Paskaranandavadivel N. O'Grady G. Tang S. J. Cheng L. K. (2015b). A theoretical study of the initiation, maintenance and termination of gastric slow wave re-entry. Math. Med. Biol. 32, 405–423. 10.1093/imammb/dqu023 25552487 PMC4486628

[B40] Du P. Poh Y. C. Lim J. L. Gajendiran V. O'Grady G. Buist M. L.. (2011). A preliminary model of gastrointestinal electromechanical coupling. IEEE Trans. Biomed. Eng. 58, 3491–3495. 10.1109/TBME.2011.2166155 21878406 PMC4129377

[B41] Egbuji J. U. G. O'grady Du P. Cheng L. K. Lammers W. J. Windsor J. A.. (2010). Origin, propagation and regional characteristics of porcine gastric slow wave activity determined by high-resolution mapping. Neurogastroenterol. Motil. 22, e292–300. 10.1111/j.1365-2982.2010.01538.x 20618830 PMC4110485

[B42] Familoni B. O. Kingma Y. J. Bowes K. L. (1987). Study of transcutaneous and intraluminal measurement of gastric electrical activity in humans. Med. Biol. Eng. Comput. 25, 397–402. 10.1007/BF02443360 3450990

[B43] Farrugia G. Lei S. Lin X. Miller S. M. Nath K. A. Ferris C. D.. (2003). A major role for carbon monoxide as an endogenous hyperpolarizing factor in the gastrointestinal tract. Proc. Natl. Acad. Sci. U.S.A. 100, 8567–8570. 10.1073/pnas.1431233100 12832617 PMC166269

[B44] Fullard L. Lammers W. Wake G. C. Ferrua M. J. (2014). Propagating longitudinal contractions in the ileum of the rabbit–efficiency of advective mixing. Food Funct. 5, 2731–2742. 10.1039/C4FO00487F 25000221

[B45] Gao J. Sathar S. O'Grady G. Archer R. Cheng L. K. (2015). A Stochastic algorithm for generating realistic virtual interstitial cell of Cajal networks. IEEE Trans. Biomed. Eng. 62, 2070–2078. 10.1109/TBME.2015.2412533 25781477 PMC4628824

[B46] Gharibans A. A. Kim S. Kunkel D. Coleman T. P. (2016). High-resolution electrogastrogram: *a* novel, noninvasive method for determining gastric slow-wave direction and speed. IEEE Trans. Biomed. Eng. 64, 807–815. 10.1109/TBME.2016.2579310 PMC5474202 27305668

[B47] Gizzi A. Cherubini C. Migliori S. Alloni R. Portuesi R. Filippi S. (2010). On the electrical intestine turbulence induced by temperature changes. Phys. Biol. 7:16011. 10.1088/1478-3975/7/1/016011 20147777

[B48] Gonlachanvit S. Chen Y. H. Hasler W. L. Sun W. M. Owyang C. (2003). Ginger reduces hyperglycemia-evoked gastric dysrhythmias in healthy humans: possible role of endogenous prostaglandins. J. Pharmacol. Exp. Ther. 307, 1098–1103. 10.1124/jpet.103.053421 14534370

[B49] Hasler W. L. Soudah H. C. Dulai G. Owyang C. (1995). Mediation of hyperglycemia-evoked gastric slow-wave dysrhythmias by endogenous prostaglandins. Gastroenterology 108, 727–736. 10.1016/0016-5085(95)90445-X 7875475

[B50] Huizinga J. D. Chen J. H. Zhu Y. F. Pawelka A. McGinn R. J. Bardakjian B. L.. (2014). The origin of segmentation motor activity in the intestine. Nat. Commun. 5, 3326. 10.1038/ncomms4326 24561718 PMC4885742

[B51] Kelly K. A. Code C. F. (1971). Canine gastric pacemaker. Am. J. Physiol. 220, 112–118. 10.1152/ajplegacy.1971.220.1.112 5538644

[B52] Kim J. H. Du P. Cheng L. K. (2013). Reconstruction of normal and abnormal gastric electrical sources using a potential based inverse method. Physiol. Meas. 34, 1193–1206. 10.1088/0967-3334/34/9/1193 24137714 PMC4061470

[B53] Kim M. S. Chey W. D. Owyang C. Hasler W. L. (1997). Role of plasma vasopressin as a mediator of nausea and gastric slow wave dysrhythmias in motion sickness. Am. J. Physiol. 272, G853–G862. 10.1152/ajpgi.1997.272.4.G853 9142918

[B54] Koch K. L. Stern R. M. Vasey M. Botti J. J. Creasy G. W. Dwyer A. (1990). Gastric dysrhythmias and nausea of pregnancy. Dig. Dis. Sci. 35, 961–968. 10.1007/BF01537244 2384042

[B55] Komorowski D. Pietraszek S. (2016). The Use of Continuous wavelet transform based on the fast fourier transform in the analysis of multi-channel electrogastrography recordings. J. Med. Syst. 40, 10. 10.1007/s10916-015-0358-4 26573647 PMC4646928

[B56] Komorowski D. Tkacz E. (2015). A new method for attenuation of respiration artifacts in electrogastrographic (EGG) signals. Conf. Proc. IEEE Eng. Med. Biol. Soc. 2015, 6006–6009. 10.1109/EMBC.2015.7319760 26737660

[B57] Kurahashi M. Nakano Y. Hennig G. W. Ward S. M. Sanders K. M. (2012). Platelet-derived growth factor receptor alpha-positive cells in the tunica muscularis of human colon. J. Cell. Mol. Med. 16, 1397–1404. 10.1111/j.1582-4934.2011.01510.x 22225616 PMC3477549

[B58] Lammers W. J. (2013). Arrhythmias in the gut. Neurogastroenterol. Motil. 25, 353–357. 10.1111/nmo.12116 23490042

[B59] Lammers W. J. el-Kays A. Manefield G. W. Arafat K. el-Sharkawy T. Y. (1997). Disturbances in the propagation of the slow wave during acute local ischaemia in the feline small intestine. Eur. J. Gastroenterol. Hepatol. 9, 381–388. 10.1097/00042737-199704000-00012 9160202

[B60] Lammers W. J. Michiels B. Voeten J. Ver Donck L. Schuurkes J. A. (2008a). Mapping slow waves and spikes in chronically instrumented conscious dogs: automated on-line electrogram analysis. Med. Biol. Eng. Comput. 46, 121–129. 10.1007/s11517-007-0294-7 18200451

[B61] Lammers W. J. Stephen B. (2008). Origin and propagation of individual slow waves along the intact feline small intestine. Exp. Physiol. 93, 334–346. 10.1113/expphysiol.2007.039180 18156170

[B62] Lammers W. J. Stephen B. Karam S. M. (2012). Functional reentry and circus movement arrhythmias in the small intestine of normal and diabetic rats. Am. J. Physiol. Gastrointest. Liver Physiol. 302, G684–G689. 10.1152/ajpgi.00332.2011 22207580

[B63] Lammers W. J. Stephen B. S. Karam S. M. (2015). Slow wave dysrhythmias in the diabetic small intestine. Neurogastroenterol. Motil. 27, 1344. 10.1111/nmo.12608 26303049

[B64] Lammers W. J. Ver Donck L. Stephen B. Smets D. Schuurkes J. A. (2008b). Focal activities and re-entrant propagations as mechanisms of gastric tachyarrhythmias. Gastroenterology 135, 1601–1611. 10.1053/j.gastro.2008.07.020 18713627

[B65] Leahy A. Besherdas K. Clayman C. Mason I. Epstein O. (1999). Abnormalities of the electrogastrogram in functional gastrointestinal disorders. Am. J. Gastroenterol. 94, 1023–1028. 10.1111/j.1572-0241.1999.01007.x 10201477

[B66] Leahy A. Besherdas K. Clayman C. Mason I. Epstein O. (2001). Gastric dysrhythmias occur in gastro-oesophageal reflux disease complicated by food regurgitation but not in uncomplicated reflux. Gut 48, 212–215. 10.1136/gut.48.2.212 11156642 PMC1728202

[B67] Lees-Green R. Du P. O'Grady G. Beyder A. Farrugia G. Pullan A. J. (2011). Biophysically based modeling of the interstitial cells of cajal: current status and future perspectives. Front. Physiol. 2:29. 10.3389/fphys.2011.00029 21772822 PMC3131535

[B68] Lien H. C. Sun W. M. Chen Y. H. Kim H. Hasler W. Owyang C. (2003). Effects of ginger on motion sickness and gastric slow-wave dysrhythmias induced by circular vection. Am. J. Physiol. Gastrointest. Liver Physiol. 284, G481–G489. 10.1152/ajpgi.00164.2002 12576305

[B69] Lim H. C. Lee S. I. Chen J. D. Park H. (2012). Electrogastrography associated with symptomatic changes after prokinetic drug treatment for functional dyspepsia. World J. Gastroenterol. 18, 5948–5956. 10.3748/wjg.v18.i41.5948 23139612 PMC3491603

[B70] Lin X. Chen J. Z. (2001). Abnormal gastric slow waves in patients with functional dyspepsia assessed by multichannel electrogastrography. Am. J. Physiol. Gastrointest. Liver Physiol. 280, G1370–G1375. 10.1152/ajpgi.2001.280.6.G1370 11352832

[B71] Lin Z. Y. McCallum R. W. Schirmer B. D. Chen J. D. (1998). Effects of pacing parameters on entrainment of gastric slow waves in patients with gastroparesis. Am. J. Physiol. 274, G186–G191. 10.1152/ajpgi.1998.274.1.G186 9458788

[B72] Linkens D. A. (1978). Canine colonic pacing and coupled oscillator synchronization [proceedings]. J. Physiol. 278, 26P-27P. 671296

[B73] Linkens D. A. Mhone P. G. (1979). Frequency transients in a coupled oscillator model of intestinal myoelectrical activity. Comput. Biol. Med. 9, 131–144. 10.1016/0010-4825(79)90029-5 455937

[B74] Lloyd C. M. Halstead M. D. Nielsen P. F. (2004). CellML: its future, present and past. Prog. Biophys. Mol. Biol. 85, 433–450. 10.1016/j.pbiomolbio.2004.01.004 15142756

[B75] Maltsev A. V. Maltsev V. A. Stern M. D. (2017). Clusters of calcium release channels harness the Ising phase transition to confine their elementary intracellular signals. Proc. Natl. Acad. Sci. U.S.A. 114, 7525–7530. 10.1073/pnas.1701409114 28674006 PMC5530665

[B76] Malysz J. Gibbons S. J. Saravanaperumal S. A. Du P. Eisenman S. T. Cao C.. (2017). Conditional genetic deletion of Ano1 in interstitial cells of Cajal impairs Ca^2+^ transients and slow waves in adult mouse small intestine. Am. J. Physiol. Gastrointest. Liver Physiol. 312, G228–G245. 10.1152/ajpgi.00363.2016 27979828 PMC5401988

[B77] Marriott H. J. (1984). Arrhythmia versus dysrhythmia. Am. J. Cardiol. 53, 628. 10.1016/0002-9149(84)90043-2 6695794

[B78] McNearney T. A. Sallam H. S. Hunnicutt S. E. Doshi D. Wollaston D. E. Mayes M. D.. (2009). Gastric slow waves, gastrointestinal symptoms and peptides in systemic sclerosis patients. Neurogastroenterol. Motil. 21, 1269–e120. 10.1111/j.1365-2982.2009.01350.x 19566588 PMC3176740

[B79] Nelsen T. S. Kohatsu S. (1968). Clinical electrogastrography and its relationship to gastric surgery. Am. J. Surg. 116, 215–222. 10.1016/0002-9610(68)90496-0 5675278

[B80] O'Grady G. Angeli T. R. Du P. Lahr C. Lammers W. J. Windsor J. A.. (2012a). Abnormal initiation and conduction of slow-wave activity in gastroparesis, defined by high-resolution electrical mapping. Gastroenterology 143, 589-98 e1–e3. 10.1053/j.gastro.2012.05.036 22643349 PMC3429650

[B81] O'Grady G. Du P. Cheng L. K. Egbuji J. U. Lammers W. J. Windsor J. A.. (2010a). Origin and propagation of human gastric slow-wave activity defined by high-resolution mapping. Am. J. Physiol. Gastrointest. Liver Physiol. 299, G585–G592. 10.1152/ajpgi.00125.2010 20595620 PMC2950696

[B82] O'Grady G. Du P. Lammers W. J. Egbuji J. U. Mithraratne P. Chen J. D.. (2010b). High-resolution entrainment mapping of gastric pacing: a new analytical tool. Am. J. Physiol. Gastrointest. Liver Physiol. 298, G314–G321. 10.1152/ajpgi.00389.2009 19926815 PMC2822498

[B83] O'Grady G. Du P. Paskaranandavadivel N. Angeli T. R. Lammers W. J. Asirvatham S. J.. (2012b). Rapid high-amplitude circumferential slow wave propagation during normal gastric pacemaking and dysrhythmias. Neurogastroenterol. Motil. 24, e299–e312. 10.1111/j.1365-2982.2012.01932.x 22709238 PMC3383091

[B84] O'Grady G. Paskaranandavadivel N. Du P. Angeli T. Erickson J. C. Cheng L. K. (2017). Correct techniques for extracellular recordings of electrical activity in gastrointestinal muscle. Nat. Rev. Gastroenterol. Hepatol. 14, 372. 10.1038/nrgastro.2017.15 28356583

[B85] O'Grady G. Wang T. H. Du P. Angeli T. Lammers W. J. Cheng L. K. (2014). Recent progress in gastric arrhythmia: pathophysiology, clinical significance and future horizons. Clin. Exp. Pharmacol. Physiol. 41, 854–862. 10.1111/1440-1681.12288 25115692 PMC4359928

[B86] Ordog T. Takayama I. Cheung W. K. Ward S. M. Sanders K. M. (2000). Remodeling of networks of interstitial cells of Cajal in a murine model of diabetic gastroparesis. Diabetes 49, 1731–1739. 10.2337/diabetes.49.10.1731 11016458

[B87] Owyang C. Hasler W. L. (2002). Physiology and pathophysiology of the interstitial cells of Cajal: from bench to bedside. VPathogenesis, I and therapeutic approaches to human gastric dysrhythmias. Am. J. Physiol. Gastrointest. Liver Physiol. 283, G8–G15. 10.1152/ajpgi.00095.2002 12065286

[B88] Parkman H. P. Hasler W. L. Barnett J. L. Eaker E. Y. (2003). Electrogastrography: a document prepared by the gastric section of the American motility society clinical GI motility testing task force. Neurogastroenterol. Motil. 15, 89–102. 10.1046/j.1365-2982.2003.00396.x 12680908

[B89] Parsons S. P. Huizinga J. D. (2016). Spatial noise in coupling strength and natural frequency within a pacemaker network; consequences for development of intestinal motor patterns according to a weakly coupled phase oscillator model. Front. Neurosci. 10:19. 10.3389/fnins.2016.00019 26869875 PMC4740389

[B90] Parsons S. P. Huizinga J. D. (2017). The phase response and state space of slow wave contractions in the small intestine. Exp. Physiol. 102, 1118–1132. 10.1113/EP086373 28671737

[B91] Paskaranandavadivel N. Cheng L. K. Du P. Rogers J. M. O'Grady G. (2017). High-resolution mapping of gastric slow wave recovery profiles: biophysical model, methodology and demonstration of applications. Am. J. Physiol. Gastrointest. Liver Physiol. 313, G265–G276. 10.1152/ajpgi.00127.2017 28546283

[B92] Paskaranandavadivel N. O'Grady G. Du P.. (2013). Comparison of filtering methods for extracellular gastric slow wave recordings. Neurogastroenterol. Motil. 25, 79–83. 10.1111/nmo.12012 22974243 PMC3535517

[B93] Pfaffenbach B. Adamek R. J. Bartholomaus C. Wegener M. (1997). Gastric dysrhythmias and delayed gastric emptying in patients with functional dyspepsia. Dig. Dis. Sci. 42, 2094–2099. 10.1023/A:1018826719628 9365141

[B94] Pitt-Francis J. Bernabeu M. O. Cooper J. Garny A. Momtahan L. Osborne J.. (2008). Chaste: using agile programming techniques to develop computational biology software. Philos. Trans. A Math. Phys. Eng. Sci. 366, 3111–3136. 10.1098/rsta.2008.0096 18565813

[B95] Plank G. Burton R. A. Hales P. Bishop M. Mansoori T. Bernabeu M. O.. (2009). Generation of histo-anatomically representative models of the individual heart: tools and application. Philos. Trans. A Math. Phys. Eng. Sci. 367, 2257–2292. 10.1098/rsta.2009.0056 19414455 PMC2881535

[B96] Poh Y. C. Beyder A. Strege P. R. Farrugia G. Buist M. L. (2012). Quantification of gastrointestinal sodium channelopathy. J. Theor. Biol. 293, 41–48. 10.1016/j.jtbi.2011.09.014 21959314 PMC3524340

[B97] Potse M. Vinet A. Opthof T. Coronel R. (2009). Validation of a simple model for the morphology of the T wave in unipolar electrograms. Am. J. Physiol. Heart Circ. Physiol. 297, H792–801. 10.1152/ajpheart.00064.2009 19465555

[B98] Sachse F. B. Moreno A. P. Seemann G. Abildskov J. A. (2009). A model of electrical conduction in cardiac tissue including fibroblasts. Ann. Biomed. Eng. 37, 874–889. 10.1007/s10439-009-9667-4 19283480

[B99] Sanders K. M. Ward S. M. Hennig G. W. (2016). Problems with extracellular recording of electrical activity in gastrointestinal muscle. Nat. Rev. Gastroenterol. Hepatol. 13, 731–774. 10.1038/nrgastro.2016.161 27756919 PMC8325940

[B100] Sathar S. Trew M. L. Du P. O'Grady G. Cheng L. K. (2014). A biophysically based finite-state machine model for analyzing gastric experimental entrainment and pacing recordings. Ann. Biomed. Eng. 42, 858–870. 10.1007/s10439-013-0949-5 24276722 PMC3972386

[B101] Sathar S. Trew M. L. O. G. G. Cheng L. K. (2015). A Multiscale tridomain model for simulating bioelectric gastric pacing. IEEE Trans. Biomed. Eng. 62, 2685–2692. 10.1109/TBME.2015.2444384 26080372 PMC4655104

[B102] Schaap H. M. Smout A. J. Akkermans L. M. (1990). Myoelectrical activity of the Billroth II gastric remnant. Gut 31, 984–988. 10.1136/gut.31.9.984 2210466 PMC1378652

[B103] Simonian H. P. Panganamamula K. Chen J. Z. Fisher R. S. Parkman H. P. (2004). Multichannel electrogastrography (EGG) in symptomatic patients: a single center study. Am. J. Gastroenterol. 99, 478–485. 10.1111/j.1572-0241.2004.04103.x 15056089

[B104] Singh R. D. Gibbons S. J. Saravanaperumal S. A. Du P. Hennig G. W. Eisenman S. T.. (2014). Ano1, a Ca^2+^-activated Cl- channel, coordinates contractility in mouse intestine by Ca^2+^ transient coordination between interstitial cells of Cajal. J. Physiol. 592, 4051–4068. 10.1113/jphysiol.2014.277152 25063822 PMC4198014

[B105] Somarajan S. Muszynski N. D. Cheng L. K. Bradshaw L. A. Naslund T. C. Richards W. O. (2015). Noninvasive biomagnetic detection of intestinal slow wave dysrhythmias in chronic mesenteric ischemia. Am. J. Physiol. Gastrointest. Liver Physiol. 309, G52–G58. 10.1152/ajpgi.00466.2014 25930082 PMC4491509

[B106] Stern R. M. Koch K. L. Stewart W. R. Lindblad I. M. (1987). Spectral analysis of tachygastria recorded during motion sickness. Gastroenterology 92, 92–97. 10.1016/0016-5085(87)90843-2 3781204

[B107] Szurszewski J. H. (1998). A 100-year perspective on gastrointestinal motility. Am. J. Physiol. 274, G447–G453. 10.1152/ajpgi.1998.274.3.G447 9565541

[B108] Szurszewski J. H. Farrugia G. (2004). Carbon monoxide is an endogenous hyperpolarizing factor in the gastrointestinal tract. Neurogastroenterol. Motil. 16(Suppl. 1), 81–85. 10.1111/j.1743-3150.2004.00480.x 15066010

[B109] Trommer P. R. (1982). Cardiolocution and dysrhythmia. Am. J. Cardiol. 50, 1198. 10.1016/0002-9149(82)90445-3 7137044

[B110] van Helden D. F. Laver D. R. Holdsworth J. Imtiaz M. S. (2010). Generation and propagation of gastric slow waves. Clin. Exp. Pharmacol. Physiol. 37, 516–524. 10.1111/j.1440-1681.2009.05331.x 19930430

[B111] Wei R. Parsons S. P. Huizinga J. D. (2017). Network properties of interstitial cells of Cajal affect intestinal pacemaker activity and motor patterns, according to a mathematical model of weakly coupled oscillators. Exp. Physiol. 102, 329–346. 10.1113/EP086077 28036151

[B112] Yeoh J. W. Corrias A. Buist M. L. (2016). A mechanistic model of a PDGFRalpha(+) cell. J. Theor. Biol. 408, 127–136. 10.1016/j.jtbi.2016.08.004 27521526

[B113] Yeoh J. W. Corrias A. Buist M. L. (2017). Modelling human colonic smooth muscle cell electrophysiology. Cell. Mol. Bioeng. 10, 186–197. 10.1007/s12195-017-0479-6 PMC6816779 31719859

